# Genetics of osteoporosis: searching for candidate genes for bone fragility

**DOI:** 10.1590/2359-3997000000178

**Published:** 2016-08-01

**Authors:** Manuela G. M. Rocha-Braz, Bruno Ferraz-de-Souza

**Affiliations:** 1 Hospital das Clínicas Faculdade de Medicina Universidade de São Paulo São Paulo SP Brasil Divisão de Endocrinologia e Laboratório de Investigação Médica 18 (LIM-18), Hospital das Clínicas da Faculdade de Medicina da Universidade de São Paulo (HCFMUSP), São Paulo, SP, Brasil; 2 Irmandade da Santa Casa de Misericórdia de São Paulo São Paulo SP Brasil Endocrinologia, Irmandade da Santa Casa de Misericórdia de São Paulo (ISCMSP), São Paulo, SP, Brasil

**Keywords:** GWAS, mutation, fracture, low bone mass, bone remodeling

## Abstract

The pathogenesis of osteoporosis, a common disease with great morbidity and mortality, comprises environmental and genetic factors. As with other complex disorders, the genetic basis of osteoporosis has been difficult to identify. Nevertheless, several approaches have been undertaken in the past decades in order to identify candidate genes for bone fragility, including the study of rare monogenic syndromes with striking bone phenotypes (*e.g.* osteogenesis imperfecta and osteopetroses), the analysis of individuals or families with extreme osteoporotic phenotypes (*e.g.* idiopathic juvenile and pregnancy-related osteoporosis), and, chiefly, genome-wide association studies (GWAS) in large populations. Altogether, these efforts have greatly increased the understanding of molecular mechanisms behind bone remodelling, which has rapidly translated into the development of novel therapeutic strategies, exemplified by the tales of cathepsin K (*CTSK*) and sclerostin (*SOST*). Additional biological evidence of involvement in bone physiology still lacks for several candidate genes arisen from GWAS, opening an opportunity for the discovery of new mechanisms regulating bone strength, particularly with the advent of high-throughput genomic technologies. In this review, candidate genes for bone fragility will be presented in comprehensive tables and discussed with regard to how their association with osteoporosis emerged, highlighting key players such as *LRP5, WNT1* and *PLS3*. Current limitations in our understanding of the genetic contribution to osteoporosis, such as yet unidentified genetic modifiers, may be overcome in the near future with better genotypic and phenotypic characterisation of large populations and the detailed study of candidate genes in informative individuals with marked phenotype.

## INTRODUCTION

Osteoporosis is a common disease characterized by low bone mineral density (BMD) and microarchitectural deterioration, leading to increased fracture risk with great morbidity and mortality, resulting in social and economic burden (1,2). Clinical diagnosis of osteoporosis is established by assessing BMD by dual-energy X-ray absorptiometry (DXA), a predictor of fracture risk, or by the occurrence of fragility fractures (3,4).

Osteoporosis is a complex disorder, influenced by both environmental and genetic factors. In the study of complex disorders, the genetic influence can be inferred from estimations of heritability, i.e., the portion of phenotypic variance attributable to cumulative genetic factors (5). In osteoporosis, BMD heritability has been estimated from 50 to 85% and, more variably, fracture heritability has ranged from 25 to 68% (6,7). Supporting the intuitive concept that the genetic influence should be more pronounced in cases of early or idiopathic osteoporosis, fracture heritability is higher for fractures occurring before 70 years of age (8).

The identification of human genes associated with bone fragility started around the 1990s through the study of monogenic syndromes with marked skeletal phenotypes such as osteogenesis imperfecta due to *COL1A1* and *COL1A2* defects (9) and osteopetrosis due to *TCIRG1* defects (10). In 2001, the breakthrough discovery of the involvement of the Wnt signalling pathway on the regulation of bone remodelling was made possible by the study of rare conditions such as osteoporosis-pseudoglioma syndrome (OPPG) due to *LRP5* mutations (11) and sclerosteosis due to *SOST* defects (12,13). More recently, the study of subjects with extreme phenotypes of osteoporosis, such as idiopathic juvenile osteoporosis and pregnancy-associated osteoporosis has yielded *WNT1* and *PLS3* as novel regulators of bone strength (14-16).

The advent of genome-wide association studies (GWAS) expanded the horizon of the genetic contribution to osteoporosis. Following a proof of concept study in 2007 (17), two pioneer GWAS for BMD were published in 2008 (18,19), identifying five significant loci associated with BMD, four of them near genes already known or suspected to be involved in the pathophysiology of osteoporosis (*RANKL*, *OPG*, *ESR1*, *LRP5*). Highlighting the potential of GWAS for gene discovery, the remaining locus mapped to novel candidate gene *ZBTB40*, later confirmed by subsequent analyses (20). Since then, more than twenty GWAS have been performed interrogating genetic association to BMD, quantitative ultrasound and/or fracture, implicating more than 90 candidate genes for osteoporosis. The function of some of these genes in bone metabolism was only recognized following their identification by GWAS (for example, *AXIN1* and *WLS*), but for the majority of candidates a biological mechanism remains unknown (7).

The identification of molecular pathways in osteoporosis has important implications not only for the recognition of individuals in risk, aiming for a personalized medical approach, but also for the development of new therapeutic strategies, as exemplified by the advent of sclerostin inhibition as a potential treatment for osteoporosis roughly ten years after the identification of *SOST* defects (21). Considering the fast paced evolution in the field, it is important to gather genetic factors involved with osteoporosis from multiple experimental sources and revise them in light of their contribution to our pathophysiological insight. In this review, a thorough and up-to-date list of candidate genes for bone fragility will be presented and discussed according to how they emerged: from rare monogenic diseases with high impact on bone strength, from extreme phenotypes of osteoporosis and/or from GWAS.

## LITERATURE SEARCH STRATEGY

**Figure 1:**
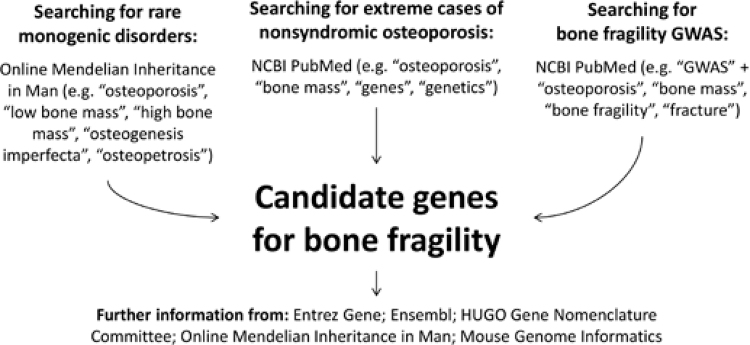
Scheme of the literature search strategy devised in order to identify candidate genes for bone fragility from rare monogenic phenotypes, extreme nonsyndromic cases of osteoporosis and genome wide association studies (GWAS) with bone fragility endpoints.

In order to identify genes associated with bone fragility, a broad literature search strategy was devised (Figure 1). A systematic review of original and review articles indexed on PubMed published until October 2015 using the descriptors “osteoporosis”, “genes”, “genetics”, and “bone mass” was undertaken. To retrieve all GWAS on bone fragility, search queries “GWAS and osteoporosis”, “GWAS and fractures”, “GWAS and bone fragility”, and “GWAS and BMD” were used. To enhance our discovery of monogenic disorders associated with altered bone mass or strength, the Online Mendelian Inheritance in Man^®^ (OMIM^®^) database was also searched using standard descriptors. Mouse phenotypic data for identified candidate genes were obtained from the Mouse Genome Informatics (MGI) online database, and gene function information was searched on NCBI’s Entrez Gene database.

## CANDIDATE GENES EMERGING FROM RARE MONOGENIC DISORDERS

The study of monogenic diseases with high impact on bone strength has enabled the identification of several pivotal mechanisms involved in bone physiology (22). For example, osteogenesis imperfecta has shown the importance of bone collagen matrix quality; Van Buchem disease, Hajdu-Cheney syndrome and autosomal recessive osteopetrosis have revealed important signalling pathways (namely Wnt, Notch and RANK-RANKL-OPG) that regulate bone remodelling; and pycnodysostosis has given insight into the pivotal action of cathepsin K in osteoclast function. On par with a recently proposed taxonomy of rare genetic disorders of bone metabolism (22), monogenic diseases will be presented according to how they affect bone strength. Candidate genes for bone fragility arising from these disorders are presented in Table 1.

**Table 1:** Genes associated with rare monogenic diseases with high impact on bone mass/strength

Gene	OMIM id	Protein function	Disease	Phenotype
*COL1A1*	120150	Type 1 collagen	Osteogenesis imperfecta	Low BMD and increased fracture risk; severity varies from perinatal lethality to asymptomatic; extra-skeletal features include blue sclerae, dentinogenesis imperfecta and hearing loss
*COL1A2*	120160	Type 1 collagen		
*BMP1*	112264	C-propeptide cleavage		
*CRTAP*	605497	Collagen hydroxylation		
*FKBP10*	607063	Collagen processing		
*IFITM5*	614757	Mineralization		
*P3H1*	610339	Collagen hydroxylation		
*PLS3*	300131	Actin-binding		
*PPIB*	123841	Collagen hydroxylation		
*SEC24D*	607186	ER procollagen processing		
*SERPINF1*	172860	Collagen chaperoning		
*SERPINH1*	600943	Mineralization		
*SP7*	606633	Ob regulation		
*TMEM38B*	611236	Cation channel		
*WNT1*	164820	Ob activation/Wnt signalling (ligand)		
*SEC24D*	607186	ER procollagen processing	Cole-carpenter syndrome	Bone fragility; craniosynostosis; ocular proptosis; hydrocephalus; distinctive facial features
*P4HB*	176790	ER procollagen processing		
*FKBP10*	607063	Collagen processing	Bruck syndrome	Congenital contractures; early onset of fractures; short stature; severe limb deformity; progressive scoliosis
*PLOD2*	601865	ER procollagen processing		
*TCIRG1*	604592	Oc function	Osteopetrosis	High BMD; skeletal deformities; compression of noble structures and occupation of bone marrow space; variable severity and age of onset
*CLCN7*	602727	Oc function		
*OSTM1*	607649	Oc homeostasis		
*PLEKHM1*	611466	Oc function		
*CA2*	611492	Oc function		
*SNX10*	614780	Oc homeostasis		
*TNFRSF11A*	603499	Oc activation (RANK)		
*TNFSF11*	602642	Oc activation (RANKL)		
*CTSK*	601105	Oc function	Pycnodysostosis	Short stature; skull deformities; acroosteolysis; high BMD; increased fracture risk
*SOST*	605740	Ob activation/Wnt signalling (antagonist)	Sclerosteosis, van Buchem disease	High BMD; increased bone strength; increased head circumference; compression of noble structures; enlarged mandible; syndactyly; high stature
*LRP5*	603506	Ob activation/Wnt signalling (receptor)	High bone mass syndrome	High BMD; increased bone strength; widened mandible; torus palatinus
			Osteoporosis-pseudoglioma	Early-onset osteoporosis; ocular pseudoglioma or vitreoretinopathy
*NOTCH2*	600275	Notch signalling	Hajdu-Cheney syndrome	Osteoporosis; short stature; acroosteolysis; distinctive facial features

Proven or proposed protein functions are shown. Ob: osteoblast; Oc: osteoclast; OMIM id: online Mendelian inheritance in men identifier; ER: endoplasmic reticulum; RANK: receptor activator of nuclear factor kappa-β; RANKL: RANK ligand.

### Monogenic diseases affecting the bone matrix

Osteogenesis imperfecta (OI) is a systemic disease characterized by high incidence of low-trauma fractures since birth or childhood due to defects in the bone matrix, chiefly in the quantity or quality of type I collagen (23,24). Clinical presentation is highly heterogeneous, with severity ranging from perinatal lethality to mostly asymptomatic. Extraskeletal features, such as blue sclerae, defective tooth development and hearing loss as well as family history may be present, allowing for an easier diagnosis. When none of these features are present, diagnosing OI can be challenging due to the overlap with idiopathic osteoporosis. Most commonly, OI is an autosomal dominant condition caused by mutations in *COL1A1* and *COL1A2* leading to clinical forms I to IV (25). Type V OI has recently been shown to be caused by mutations in *IFITM5*, also transmitted in an autosomal dominant pattern; the exact role of IFITM5 in determining bone strength remains elusive (26,27). Several rarer forms of OI with autosomal recessive inheritance exist, and the list of candidate genes for such phenotypes is ever increasing (Table 1). Most genes associated with recessive OI are directly or indirectly involved with type I collagen modification and/or assembly, but for some a mechanism is still unknown (28). Collectively, OI demonstrates how defects in bone material properties may have a substantial impact on bone strength.

More than 400 genetic skeletal disorders have been described, with around 360 genes implicated (29). A number of these skeletal dysplasias may also lead to bone fragility. In particular, Bruck syndrome and Cole-Carpenter syndrome have marked fragility, and their heterogeneous genetic bases overlap with OI (Table 1). Bruck syndrome, characterised by congenital joint contractures and early onset of fractures, can be caused by mutations in *FKBP10* or *PLOD2*, and Cole-Carpenter syndrome, characterised by bone fragility, craniosynostosis and distinctive facies, has been associated with *P4HB* and *SEC24D* defects. Mutations in *FKBP10* and *SEC24D* have also been implicated in OI, meaning that variants with variable biological impact may have different phenotypic expression and lead to isolated bone fragility (30,31).

### Monogenic diseases affecting bone remodelling

Impairment of osteoclast-mediated bone resorption is known to lead to high bone mass syndromes such as osteopetrosis and pycnodysostosis (Table 1). In spite of the high bone mass, a high fracture risk is usually observed due to impaired bone renewal leading to poor quality.

Osteopetrosis is characterized by skeletal deformities, nerve compression and bone marrow occupation, and may present with variable degree of severity and inheritance patterns. Defects in the RANK-RANKL-OPG pathway, pivotal to osteoclast differentiation and activation, lead to autosomal recessive osteopetrosis due to a reduced number of osteoclasts (32). In contrast, defects in several genes involved in osteoclast function may lead to osteopetrosis with a normal or high number of osteoclasts. Of note, mutations in *CLCN7*, *CA2* and *TCIRG1,* disrupting the regulation of organelle pH and acid secretion, may cause osteopetrosis by affecting the osteoclast ability to dissolve the bone matrix (32).

Pycnodysostosis, marked by high bone mass, short stature, skull deformities and acroosteolysis, is caused by mutations in *CTSK* encoding cathepsin K, an enzyme secreted by osteoclasts and crucial to bone resorption (33). The identification of *CTSK* defects as the cause of pycnodysostosis in 1996, and subsequent studies of its function in bone resorption, has led to the development of cathepsin K inhibition as a promising therapeutic approach for osteoporosis 20 years later, highlighting the importance of recognising molecular mechanisms in order to advance medical care and the fast pace of translation in this burgeoning field (34).

Disruption in bone formation may lead to either low BMD, and consequently decreased bone strength, or may inversely cause abnormally high BMD, with stronger bone and possibly decreased risk of fracture. Defects in members of the Wnt signalling pathway, key to osteoblast activation and function, illustrate how these opposite phenotypes might ensue (35). Activation of the Wnt receptor LRP5 ultimately leads to increased beta-catenin and osteoblast activity. Inactivating mutations in *LRP5* lead to osteoporosis-pseudoglyoma syndrome, characterized by severe early-onset osteoporosis and ocular malformation, whereas gain-of-function *LRP5* mutations (which abolish interaction with inhibitors Dkk-1 and sclerostin) lead to the high bone mass syndrome endosteal hyperostosis (Worth disease) (11,36). Accordingly, loss of the bone-specific Wnt inhibitor sclerostin (SOST) due to inactivating *SOST* mutations or deletion of its regulatory region lead to sclerosteosis and Van Buchem disease, marked by high BMD with skeletal deformities such as jaw and cranial enlargement (12,13,37). The painstaking study of these rare disorders led to recognition of sclerostin’s crucial repressive role in bone formation; its inhibition is currently being investigated in the treatment of osteoporosis in randomised clinical trials and may represent a paradigm shift in osteoporosis care in the near future (21).

Finally, Hajdu-Cheney syndrome, a rare form of syndromic osteoporosis accompanied by coarse and dysmorphic facies, short stature and acroosteolysis, is caused by *NOTCH2* mutations disrupting Notch signalling (38,39). While the molecular physiology of Notch signalling in bone is still incompletely understood, the pronounced bone fragility in Hajdu-Cheney syndrome underlines the opportunity for novel therapeutic strategies targeting this pathway.

It should be noted that genetic defects associated with osteomalacia, primarily relating to bone mineralization, may also lead to osteoporosis-like milder phenotypes characterized by fragility fractures; within this vast group of disorders, attention is currently drawn to heterozygous *ALPL* mutations leading to adult hypophosphatasia, which has been proposed to be a potentially under-recognised cause of bone fragility (40).

## CANDIDATE GENES IDENTIFIED THROUGH EXTREME CASES OF OSTEOPOROSIS

The quest for the genetic basis of a few extreme cases of nonsyndromic idiopathic osteoporosis has been reported in the literature. In general, a candidate gene approach has been applied, focussing on genes associated with OI and, more recently, Wnt signalling. Even though most studies have involved small cohorts and somewhat limited genetic approaches, the advent of massively parallel sequencing is rapidly boosting our capability for establishing a molecular diagnosis in these cases. Candidate genes identified in this manner are assembled in Table 2.

**Table 2:** Genes associated with idiopathic osteoporosis

Gene	OMIM id	Function	Phenotype	Study design	Reference
*LRP5*	603506	Wnt signalling (receptor)	Juvenile osteoporosis	Candidate gene analysis (3 genes)	(43)
			Vertebral fractures during pregnancy	Candidate gene analysis (3 genes)	(46)
			Postpartum vertebral fractures	Candidate gene analysis (2 genes)	(47)
			Idiopathic juvenile osteoporosis	WES, analysis focussed on candidate genes (14 genes)	(45)
*DKK1*	605189	Wnt signalling (antagonist)	Juvenile osteoporosis	Candidate gene analysis (8 genes)	(44)
*WNT3A*	606359	Wnt signalling (ligand)	Juvenile osteoporosis	Candidate gene analysis (8 genes)	(44)
*MTHFR*	607093	Homocysteine metabolism	Postpartum vertebral fractures	Candidate gene analysis (2 genes)	(47)
*PLS3*	300131	Actin-binding protein	X-linked osteoporosis	Massively parallel sequencing strategies	(16,48,49)
*WNT1*	164820	Wnt signalling (ligand)	Early-onset autosomal dominant osteoporosis	Massively parallel sequencing strategies	(14,15)

OMIM id: online Mendelian inheritance in men identifier; WES: whole-exome sequencing.

Initially, well known OI genes *COL1A1* and *COL1A2* posed as conspicuous candidates for mutational analysis in individuals with bone fragility. In 1991, Spotila and cols. investigated a 52-yo postmenopausal woman with low bone mass and a vertebral fracture, identifying a *COL1A2* mutation (41). Of note, this patient had mildly blue sclerae and mild hearing loss, suggesting a mild presentation of OI. In 1994, the same group of authors undertook a mutational analysis of *COL1A1* and *COL1A2* in a cohort of 26 individuals with low bone density, identifying other two mutations in *COL1A1* in association with milder phenotypes (42).

As novel molecular mechanisms in bone fragility were recognised, further genes became candidates for investigation. In 2005 and 2012, Hartikka and cols. and Korvala and cols. reported the mutational analysis of a cohort of children with idiopathic osteoporosis, examining a total of 11 candidate genes mainly associated with autosomal dominant OI or the Wnt signalling pathway (43,44). Initially, Hartikka and cols. studied *COL1A1*, *COL1A2* and *LRP5*, identifying three distinct mutations in *LRP5* in 3 children, with some evidence of familial segregation (43). Later, Korvala and cols. studied 8 new candidate genes, and identified rare sequence variants in two children (44). In one subject they found a heterozygous missense variant in *WNT3A*, which was also present in an affected sister, inherited from their mother who presented with post-menopausal osteoporosis. Nonetheless, the paternal family, who did not carry this variant, had a prominent history of adult osteoporosis and fractures, suggesting that other genetic factors might also be associated with the more severe/early-onset phenotype. In the other subject, a rare variant in *DKK1*, a well-known inhibitor of Wnt signalling, was identified, albeit with incomplete segregation (44).

Further studies have associated *LRP5* variants with an array of extreme osteoporosis phenotypes (Table 2). Also using a candidate gene approach, Fahiminiya and cols., Campos-Obando and cols., and Cook and cols. have studied single cases and found three different *LRP5* variants in two women with pregnancy-related osteoporosis and vertebral fractures, and one boy with idiopathic juvenile osteoporosis (45-47). Segregation analyses did not show clear relationships between variants and phenotype, again suggesting the association of additional genetic and/or environmental factors. One of the subjects with pregnancy-related osteoporosis was also homozygous for the *MTHFR* gene C677T polymorphism, which has been associated with several health outcomes including fracture risk and low BMD (47) *MTHFR* encodes for methylenetetrahydrofolate reductase, an enzyme involved in folate, homocysteine and amino acid metabolism.

The emergence of high throughput technologies allowed *de novo* discovery of candidate genes associated with familial idiopathic osteoporosis. In 2013, two groups independently identified *WNT1* mutations in this context. Keupp and cols. performed whole exome sequencing in a four-generation family with early-onset autosomal dominant osteoporosis, identifying a heterozygous *WNT1* mutation segregating with the phenotype (14). Laine and cols reported the genomewide linkage analysis followed by targeted parallel sequencing of another family with a similar presentation, also leading to the identification of a heterozygous *WNT1* mutation (15). Notably, both groups found homozygous *WNT1* mutations in families with severe recessive OI, suggesting a phenotypic spectrum of severity in relation to the molecular defects. Even though other Wnt family members were already well-known regulators of bone remodelling, these reports unravelled the importance of *WNT1* in bone strength.

The discovery of entirely novel mechanisms in bone fragility has also been made possible by massively parallel sequencing. In 2013, Van Dijk and cols. performed X-linked whole exome sequencing in a family with X-linked osteoporosis, identifying a deleterious frameshift mutation in *PLS3*, a new factor in bone metabolism (16). Four additional *PLS3* mutations were found in further four families. Notably, male individuals in these families carrying hemizygous *PLS3* variants presented with overt osteoporotic fractures while female carriers had milder phenotypes with low bone mass. Additionally, a rare *PLS3* variant (rs140121121) was found in 5 unrelated males with osteoporotic fractures and then studied in a large Dutch cohort, showing an association with increased fracture risk in elderly heterozygous female carriers, thus suggesting a role for this variant in common osteoporosis (16).

Further reports have supported a causative role for *PLS3* mutations in the genesis of X-linked osteoporosis (48,49). While the biological role of *PLS3* in bone is still largely unknown, a disturbance in osteocyte mechanosensing has been proposed as a putative mechanism based on animal model observations (16).

Taken together, these reports support a robust genetic contribution for extreme cases of osteoporosis, with potential translational implications for the care of common osteoporosis. Nevertheless, the individual impact of these variants on phenotype is still incompletely understood, and additional genetic factors may account for variable phenotypic expression in some cases.

## CANDIDATE GENES IDENTIFIED THROUGH GENOME-WIDE ASSOCIATION STUDIES (GWAS)

As with other multifactorial diseases, common osteoporosis has long been hypothesized to be caused by multiple common variants each exerting a small influence on phenotype (7). Therefore, the technological breakthrough of GWAS was wholly embraced in the field, and at least twenty-nine low BMD and/or fractures GWAS have been published since 2008, including original studies and meta-analyses. As a result, most of the genes associated with bone fragility until now have been identified through such studies, totalling more than 70 loci and, respectively, more than 90 genes, which are listed on Table 3.

**Table 3:** Genes associated with bone mineral density or fracture risk in major genome-wide association studies

Candidate gene	BMD p-value (Fracture p-value)	SNP	References
*ABCF2*	7.3x10^-9^	rs7812088	GEFOS2 [Ref. (20)]
ABL1*	3.4x10^-22^	rs7851693	GEFOS2
*ADAMTS18*	2.1x10^-8^	rs16945612	Xiong 2009 [Ref. (54)]
*ALDH7A1*	6.4x10^-6^ (2.1x10^-9^)	rs13182402	Guo 2010 [Ref. (55)]
*ANAPC1*	1.5x10^-9^	rs17040773	GEFOS2
*ARHGAP1*	5.1x10^-18^	rs7932354	GEFOS1 [Ref. (51)], GEFOS2
*ATP6V1G1*	3.0x10^-9^	rs10817638	Tan 2015 [Ref. (56)]
AXIN1*	1.0x10^-16^	rs9921222	GEFOS2
*C12orf23*	9.6x10^-10^	rs1053051	GEFOS2
*C7orf76*	8.1x10^-48^ (5.9x10^-11^)	rs4727338	GEFOS1, GEFOS2
*CCDC170*	4.0x10^-35^	rs4869742	GEFOS1, GEFOS2, Styrkarsdottir 2008 [Ref. (19)] & 2009 [Ref. (50)]
*CDC5L*	5.6x10^-11^	rs163879	GEFOS2
CLCN7*	1.5x10^-16^	rs163879	GEFOS2
*CLDN14*	4.2x10^-9^	rs170183	Zhang 2014 [Ref. (57)]
*COLEC10*	3.2x10^-39^	rs2062377	GEFOS1, GEFOS2, Styrkarsdottir 2008, Richards 2008 [Ref. (18)]
*CPED1*	6.0x10^-11^	rs13245690	GEFOS2, Zheng 2012 [Ref. (58)] & 2015 [Ref. (53)]
*CPN1*	9.0x10^-10^	rs7084921	GEFOS2
CREB3L1*	5.1x10^-18^	rs7932354	GEFOS1, GEFOS2
*CRHR1*	1.4x10^-8^	rs9303521	GEFOS1
CTNNB1*	4.4x10^-25^	rs430727	GEFOS1, GEFOS2
*CYLD*	1.9x10^-22^	rs1566045	GEFOS2
*DCDC1*	2.2x10^-11^	rs163879	GEFOS1, GEFOS2
*DCDC5*	2.2x10^-11^	rs163879	GEFOS1, GEFOS2
*DHH*	1.2x10^-15^	rs12821008	GEFOS2
DKK1*	1.6x10^-12^ (9.0x10^-9^)	rs1373004	GEFOS2
DMP1*	1.2x10^-27^ (1.7x10^-8^)	rs6532023	GEFOS2, Duncan 2011 [Ref. (52)]
*DNM3*	8.5x10^-15^	rs479336	GEFOS2
EN1*	2x10^-14^ (2x10^-11^)	rs11692564	Zheng 2015
*ERC1*	5.6x10^-12^	rs2887571	GEFOS2
ESR1*	4.0x10^-35^	rs4869742	GEFOS1, GEFOS2, Styrkarsdottir 2008 & 2009
*F2*	5.1x10^-18^	rs7932354	GEFOS1, GEFOS2
*FAM210A*	4.9x10^-8^ (8.8x10^-13^)	rs4796995	GEFOS2
*FAM3C*	1.0x10^-11^	rs7776725	Cho 2009 [Ref. (59)]
*FAM9A*	1.2x10^-8^	rs5934507	GEFOS2
*FAM9B*	1.2x10^-8^	rs5934507	GEFOS2
FKBP11*	1.2x10^-15^	rs12821008	GEFOS2
*FMN2*	1.9x10^-9^	rs9287237	Paternoster 2013 [Ref. (60)]
FOXC2*	1.0x10^-14^	rs10048146	GEFOS1, GEFOS2
*FOXL1*	1.0x10^-14^	rs10048146	GEFOS1, GEFOS2
*FUBP3*	3.4x10^-22^	rs7851693	GEFOS2
GALNT3*	4.8x10^-10^	rs6710518	Duncan 2011
*GPATCH1*	6.6x10^-11^	rs10416218	GEFOS2
GPR68*	2.0x10^-15^	rs1286083	GEFOS2
GREM2*	1.9x10^-9^	rs9287237	Paternoster 2013
*HDAC5*	1.7x10^-8^	rs228769	GEFOS1
IBSP*	1.2x10^-27^ (1.7x10^-8^)	rs6532023	Duncan 2011
*IDUA*	5.2x10^-15^	rs3755955	GEFOS2
*INSIG2*	1.2x10^-10^	rs1878526	GEFOS2
JAG1*	3.1x10^-19^	rs1878526	GEFOS2, Kung 2010 [Ref. (61)]
*KAL1*	1.2x10^-8^	rs5934507	GEFOS2
*KCNMA1*	5.0x10^-19^	rs7071206	GEFOS2
*KIAA2018*	4.1x10^-10^	rs1026364	GEFOS2
*LACTB2*	1.9x10^-8^	rs7017914	GEFOS2
*LEKR1*	4.5x10^-12^	rs344081	GEFOS2
*LGR4*	1.3x10^-10^	rs587777005	Styrkarsdottir 2013 [Ref. (62)]
*LIN7C*	4.9x10^-8^	rs10835187	GEFOS2
LRP4*	5.1x10^-18^	rs7932354	GEFOS1, GEFOS2
LRP5*	2.1x10^-26^ (1.4x10^-8^)	rs3736228	GEFOS1, GEFOS2, Kaufman 2008 [Ref. (63)]
*MARK3*	5.2x10^-16^	rs11623869	GEFOS1, GEFOS2, Sttyrkarsdottir 2009
MECOM*	3.6x10^-8^	rs784288	Hwang 2013 [Ref. (64)]
*MEF2C*	4.5x10^-61^	rs1366594	GEFOS1, GEFOS2, Duncan 2011
MEPE*	1.2x10^-27^ (1.7x10^-8^)	rs6532023	GEFOS2
*MPP7*	2.4x10^-16^	rs3905706	GEFOS2
NBR1*	2.0x10^-11^	rs4792909	GEFOS, Styrkarsdottir 2009
*NTAN1*	1.7x10^-10^	rs4985155	GEFOS2
*PDXDC1*	1.7x10^-10^	rs4985155	GEFOS2
PKDCC*	1.3x10^-9^	rs7584262	GEFOS2
PTHLH*	1.9x10^-12^	rs7953528	GEFOS2
*RPS6KA5*	2.0x10^-15^	rs1286083	GEFOS2
RSPO3*	3.0x10^-8^	rs13204965	Duncan 2011
RUNX2*	5.6x10^-11^	rs11755164	GEFOS2
SALL1*	1.9x10^-22^	rs1566045	GEFOS2
SHFM1*	8.1x10^-48^ (5.9x10^-11^)	rs4727338	GEFOS1, GEFOS2
*SLC25A13*	8.1x10^-48^ (5.9x10^-11^)	rs4727338	GEFOS2
*SMG6*	9.8x10^-19^	rs4790881	GEFOS2
SMOC1*	4.0x10^-13^	rs227425	Zhang 2014
SOST*	2.0x10^-11^	rs4792909	GEFOS2, Styrkarsdottir 2009
SOX4*	2.7x10^-13^	rs9466056	GEFOS2
SOX6*	1.1x10^-32^	rs7108738	GEFOS1, GEFOS2, Hsu 2010 [Ref. (65)]
SOX9*	1.9x10^-11^	rs7217932	GEFOS2
SP7*	3.0x10^-20^	rs2016266	GEFOS1, GEFOS2, Styrkarsdottir 2009, Timpson 2009 [Ref. (66)]
SPP1*	1.2x10^-27^ (1.7x10^-8^)	rs6532023	GEFOS2
*SPTBN1*	2.3x10^-18^ (2.6x10^-8^)	rs4233949	GEFOS1, GEFOS2
*STARD3NL*	3.8x10^-38^	rs6959212	GEFOS1, GEFOS2
SUCO*	8.5x10^-15^	rs479336	GEFOS2
*SUPT3H*	5.6x10^-11^	rs11755164	GEFOS2
TNFRSF11A (RANK)*	1.6x10^-17^	rs884205	GEFOS1, GEFOS2, Styrkarsdottir 2009
TNFRSF11B (OPG)*	3.2x10^-39^	rs2062377	GEFOS1, GEFOS2, Styrkarsdottir 2008, Richards 2008
TNFSF11 (RANKL)*	5.4x10^-25^	rs9533090	GEFOS1, GEFOS2, Styrkarsdottir 2008 & 2009
WLS*	2.6x10^-13^	rs1430742	GEFOS1, Hsu 2010, Duncan 2011
WNT16*	3.2x10^-51^	rs3801387	GEFOS2, Zheng 2012 & 2015
WNT5B*	5.6x10^-12^	rs2887571	GEFOS2
*XKR9*	1.9x10^-8^	rs7017914	GEFOS2
*ZBTB40*	7.4x10^-57^	rs6426749	GEFOS1, GEFOS2, Duncan 2011, Styrkarsdottir 2008
*ZNF408*	5.1x10^-18^	rs7932354	GEFOS1, GEFOS2

Strongest BMD/fracture p-values and corresponding single nucleotide polymorphisms (SNPs, identified according to dbSNP) are shown; only signals with a p-value less than 5x10^-8^ were included. * Indicates genes for which additional evidence of involvement in bone development and metabolism is available.

The first two major GWAS were published in 2008 by Styrkarsdottir and cols. and Richards and cols, interrogating genetic association to low BMD and low trauma fractures (18,19). Whole sample sizes comprised 13,786 and 8,557 individuals, respectively, and five major genes were identified: *OPG*, *RANKL*, *LRP5*, *ESR1* and *ZBTB40* (Table 3). As previously mentioned, OPG and RANKL regulate osteoclast differentiation and activation, and LRP5 is a crucial mediator of Wnt signalling in bone formation. *ESR1*, which encodes for the oestrogen receptor, has long been considered a candidate gene for osteoporosis, based on earlier linkage studies and oestrogens’ prominent physiological role in bone remodelling. A remaining locus identified by Styrkarsdottir and cols., rs7524102, was strongly associated with both spine and hip BMD but obvious candidate genes lacked in its vicinity. Subsequent GWAS have confirmed this locus on larger cohorts (20,50-52), with p-value reaching 7.4x10^-57^ for association with hip BMD (20). Since these signals map to an intergenic region, the association has been attributed to the closest gene, *ZBTB40*. Up to now, a biological role for *ZBTB40* in human or animal health is largely unknown.

The largest published GWAS, GEFOS2, was published in 2012 comprising data from > 80,000 subjects for BMD and > 130,000 fracture cases and controls (20). This study alone was able to identify 56 loci associated with BMD and 14 loci related to fracture risk, but still could only explain 5.8% of the genetic contribution to femoral neck BMD. These striking numbers epitomize both the great strength of GWAS in identifying genes related to common diseases and their great limitations in explaining the total genetic variability of such diseases, a concept commonly referred to as the missing heritability (5,7).

In 2015, a breakthrough GWAS based on whole-genome sequencing was published by Zheng and cols., with enough power to detect the effects of low-frequency variants (minor allele frequency [MAF] between 1-5%), which are usually not contemplated by genotype-based GWAS (53). Using this approach, the novel candidate gene *EN1* was identified*,* significantly related to both BMD and fracture risk. Animal models and *in vitro* studies indicate a possible role for *EN1* in osteoblasts, offering an exciting opportunity for the discovery of new mechanisms in bone formation (53). Finally, this study also suggests that lower frequency variants may have higher impact on BMD and fractures, warranting further studies.

A full list of the major 95 genes identified by GWAS is presented on Table 3. Remarkably, evidence of involvement in bone physiology is currently available for only 41 genes (shown in table). The remaining 54 genes were selected based on their physical proximity to the GWAS signal, and therefore their biological association to bone fragility needs to be further scrutinized.

### Future challenges

The genetics of osteoporosis have been increasingly unravelled during the past two decades. Gene defects underlying syndromic diseases with prominent skeletal phenotype have been identified, as well as genetic variants related to idiopathic and/or extreme osteoporosis. Technological advances have allowed unbiased *de novo* discovery of novel candidate genes and also of numerous loci associated to common osteoporosis. Through all these different strategies, several novel pathways regulating bone remodelling and matrix homeostasis have been recognised, pushing the boundaries of the therapeutic arsenal for bone fragility.

Concomitantly, however, gaps on our understanding of these processes have become apparent. For example, even with a great number of subjects and SNPs analysed, the largest GWAS to date can only explain 5.8% of the genetic contribution to BMD variability. Furthermore, most candidate genes or loci identified by high-throughput genomic analysis remain to have their role in bone metabolism fully elucidated. Altogether, these shortcomings pose as research challenges, warranting further exploration. In the foreseeable future, genomic analysis with enough power to detect the effects of low-frequency variants may lead to the discovery of missing heritability.

Gene defects so far identified in association with idiopathic osteoporosis are likely to have a major causative role in determining these phenotypes, but a clear genotype/phenotype correlation and precise co-segregation within families are still lacking in many cases, suggesting that a contribution of yet unfound genetic modifiers may exist. Further studies of idiopathic osteoporosis interrogating the role of candidate genes identified by GWAS for which a function in bone is still unknown might help identify such modifiers or even uncover major causative roles for some of these novel candidates. Additionally, animal models and *in vitro* studies may help to clarify their biological function in bone strength.

In conclusion, major advances in the genetics of bone fragility have allowed a deeper understanding of bone remodelling, with translational implications in many instances. Several experimental sources of candidate genes for osteoporosis have arisen, particularly due to the study of rarer informative individuals and families but also through the advent of genome-scale methods for genetic analysis. It is hoped that the continued and concerted effort of clinicians and researchers, and ongoing technological progress will further illuminate the genetic basis of osteoporosis and enable more precise treatment strategies in the near future.
